# Influence of ADHD, especially attention-deficit characteristics, on the course of alcohol-dependent individuals

**DOI:** 10.1186/s12888-022-04455-4

**Published:** 2022-12-19

**Authors:** Atsushi Yoshimura, Sachio Matsushita, Mitsuru Kimura, Jun-ichi Yoneda, Hitoshi Maesato, Akira Yokoyama, Susumu Higuchi

**Affiliations:** 1 National Hospital Organization, Kurihama Medical and Addiction Center, Yokosuka, Japan; 2 Division of Psychiatry, Tohoku Medical and Pharmaceutical University, Sendai, Japan

**Keywords:** Alcohol dependence, Clinical course, ADHD, ASRS (ADHD self-report scale), AQ (autism-spectrum quotient)

## Abstract

**Background:**

While several studies have revealed that neurodevelopmental disorders have a high probability of overlapping with substance use disorders, the effects of neurodevelopmental disorders on the courses of substance use disorders have hardly been examined.

**Methods:**

This study targeted 637 alcohol-dependent individuals who received inpatient treatment and whose drinking situations were followed for 12 months after hospital discharge using mailed questionnaires. The comorbidity of psychiatric disorders and the characteristics associated with the neurodevelopmental disorders were assessed using several measurements at the time of hospital admission. The effects of neurodevelopmental disorders on the drinking courses of the subjects were then estimated.

**Results:**

The presence of a current depressive episode or any anxiety disorder significantly lowered the abstinence rates during the follow-up period (*p* = 0.0195 and *p* = 0.0214, respectively). ADHD traits as assessed using the ADHD Self-report Scale (ASRS) predicted a significantly poorer abstinence rate (*p* = 0.0296). Similarly, attention-deficit characteristics assessed objectively through interviews predicted a significantly lower abstinence rate (*p* = 0.0346), and a sensitivity analysis enhanced these results (*p* = 0.0019). When the drinking patterns were classified into three groups, the subjects with attention-deficit characteristics had a significantly higher rate of “Recurrence” and lower rates of “Abstinence” and “Controlled drinking” (*p* = 0.013). In a multivariate proportional hazards analysis, the ASRS score was significantly correlated with the re-drinking risk (*p* = 0.003).

**Conclusion:**

ADHD traits had significant effects on not only abstinence rates, but also on drinking pattern. The presence of ADHD traits, especially attention-deficit characteristics, influenced the drinking courses of alcohol-dependent individuals after hospital treatment.

**Supplementary Information:**

The online version contains supplementary material available at 10.1186/s12888-022-04455-4.

## Introduction

Alcohol dependence may develop gradually in individuals who frequently drink alcohol habitually. The mechanisms responsible for the development of dependency can involve reward drinking or relief drinking [1, 2]. Reward drinking is mainly caused by the pharmacological effect of alcohol. Alcohol intensely affects the reward mechanism of dopaminergic neurons in the cerebral limbic system. On the other hand, relief drinking can be attributed to various factors. Since alcohol has the effect of alleviating various psychological burdens, individuals with greater psychological burdens may have a greater tendency to develop dependencies on alcohol and other substances.

Alcohol use disorder is relatively common and of significant concern among patients with severe mental disorders [3]. Subjects who meet the criteria for alcohol dependence have high rates of depression, bipolar disorder, and anxiety disorder as comorbidities [4]. The courses of alcohol-dependent individuals are thought to be influenced by psychiatric comorbidity [5–8]. Patients with alcohol use disorder who exhibit symptoms of clinical depression at either hospital admission or discharge have a significantly lower abstinence rate after inpatient treatment [6]. Patients with alcohol dependence were also more likely to have a drinking relapse during the follow-up period if they had comorbid anxiety traits or anxiety disorders [7, 8]. Thus, the presence of a psychological or psychiatric burden might influence the subsequent drinking pattern post-treatment. Some integrated treatment programs for patients with a mental disorder and co-occurring alcohol use disorder have been developed [3, 9].

Recently, neurodevelopmental disorders in adults have attracted societal attention. Adults with neurodevelopmental disorders are more likely to face social distress at home and in the workplace. They may be susceptible to substance use disorders, partly because such substances can temporarily alleviate their distress [10]. However, adolescents with autistic spectrum disorder (ASD) reportedly have lower rates of drug and alcohol misuse, compared with subjects with psychiatric disorders [11]. Another study reported that the prevalence of adults with ASD who met the lifetime criteria for substance use disorder (SUD) was 16%, which was no greater than that expected in the general population [12]. Relative to those with ASD, individuals with attention-deficit hyperactivity disorder (ADHD) are more likely to be vulnerable to substance use. The association between ADHD and the development of SUDs has been clarified [13, 14]. Both adolescents and adults with ADHD reportedly have elevated rates of comorbid SUD [15, 16]. Although several mechanisms, such as self-medication, behavioral disinhibition, novelty seeking, and sensitization effects, are assumed to be pathways to substance use among individuals with ADHD, evidence of the causal pathway has not yet been clearly delineated [17]. The presence of ADHD has been shown to predict an earlier age of onset [18], a longer duration of SUD [10], and an increased severity of SUD [13]. However, there is a dearth of research investigating the relationship between ADHD and SUD regarding treatment outcome [16].

We performed a prognostic study examining treatment-seeking alcohol-dependent patients. To examine the influence of psychological or psychiatric burden on the course of alcohol-dependent individuals, we assessed the impacts of psychiatric comorbidities and neurodevelopmental characteristics on the courses of alcohol-dependent patients after hospital treatment. The study estimated the influences in terms of abstinence rates and drinking patterns, including controlled drinking.

## Method

### Subjects

This study enrolled inpatients at the National Hospital Organization Kurihama Medical and Addiction Center, which is a central hospital for alcohol and addiction treatment in Japan. Patients who were admitted to the hospital for alcohol treatment between January 2014 and December 2014 were registered if they provided written informed consent after receiving a complete description of the study. The exclusion criterion was a score of ≤20 on the Mini Mental State Examination (MMSE) [19], indicating a cognitive function level insufficient to understand the outline of the study [20]. The subjects ranged in age from 20 to 85 years old. The baseline characteristics of the subjects according to gender and whether they were or were not followed up are shown in Table 1.

**Table 1:** Baseline characteristics of the subjects according to gender and whether they were or were not followed up

Characteristics	Mean (± SD) or Frequencies (%)					*p* value Followed vs. Not followed
	Total	Male	Female	Followed	Not followed	
Age (years)	53.9 (± 12.9)	55.2 (± 12.5)	46.2 (± 13.0)	54.2 (± 13.1)	52.9 (± 12.2)	0.322
Female	94 (14.8%)	–	–	79 (15.4%)	15 (12.0%)	0.332
Age at first drink (years)	17.5 (± 3.7)	17.3 (± 3.3)	18.7 (± 5.2)	17.5 (± 3.8)	17.2 (± 3.2)	0.336
Age at start of habitual drinking (years)	24.2 (± 8.5)	23.8 (± 8.0)	26.9 (± 10.0)	24.1 (± 8.1)	24.7 (± 9.9)	0.481
Average alcohol consumption per time (g)	124.7 (± 115.5)	127.3 (± 118.0)	108.1 (± 97.7)	122.9 (± 103.8)	132.3 ± (156.4)	0.447
Daily heavy drinking (more than 60 g)	395 (70.4%)	342 (71.0%)	53 (67.1%)	317 (70.0%)	78 (72.2%)	0.974
Alcohol dependence (ICD-10)	589 (96.1%)	509 (96.4%)	80 (94.1%)	472 (96.1%)	117 (95.9%)	0.907
History of alcohol treatment	286 (45.4%)	233 (43.4%)	53 (57.0%)	234 (46.3%)	52 (41.6%)	0.267
History of attending self-help groups	123 (20.4%)	95 (18.3%)	28 (32.9%)	103 (21.3%)	20 (16.5%)	0.268
Family history of alcohol dependence	169 (27.4%)	152 (28.8%)	17 (19.1%)	137 (27.7%)	32 (26.0%)	0.507
Smoking	479 (77.0%)	419 (79.2%)	60 (64.5%)	385 (77.3%)	94 (75.8%)	0.414
Current depressive episode (M.I.N.I.)	88 (16.2%)	75 (16.1%)	13 (16.9%)	69 (15.6%)	19 (19.0%)	0.407
Anxiety disorder (M.I.N.I.)	100 (18.4%)	76 (16.3%)	24 (31.2%)	84 (19.0%)	16 (16.0%)	0.490
Manic episode (M.I.N.I.)	35 (6.5%)	26 (5.6%)	9 (11.7%)	27 (6.1%)	8 (8.0%)	0.487
Self-rating Depression Scale (SDS)	45.7 (± 9.8)	45.2 (± 9.7)	47.8 (± 10.0)	45.4 (± 9.8)	46.6 (± 10.0)	0.300
Beck Anxiety Inventory (BAI)	8.9 (± 9.4)	8.5 (± 9.1)	10.9 (± 10.5)	8.7 (± 9.5)	9.7 (± 9.0)	0.370
ASD traits (AQ)	29 (5.9%)	23 (5.6%)	6 (7.5%)	22 (5.5%)	7 (7.7%)	0.427
ADHD traits (ASRS part A)	88 (17.1%)	71 (16.6%)	17 (20.0%)	69 (16.4%)	19 (20.4%)	0.349
Total ASRS score (ASRS part A and B)	25.3 (± 8.9)	24.9 (± 8.6)	27.3 (± 10.3)	25.2 (± 8.9)	25.7 (± 8.7)	0.641
Attention-deficit (SSAGA-II)	115 (21.1%)	96 (20.6%)	19 (24.4%)	86 (19.5%)	29 (28.2%)	0.051
Hyperactivity/Impulsivity (SSAGA-II)	102 (18.8%)	96 (20.6%)	6 (7.8%)	83 (18.9%)	19 (18.4%)	0.922

### Procedure

This prospective study was conducted over a 12-month period following inpatient alcohol treatment. The study was approved by the Ethics Committee of the National Hospital Organization Kurihama Medical and Addiction Center. In total, 712 consecutive patients were admitted for alcohol treatment during the registration period. Some patients were excluded because they had insufficient cognitive functions, refused to continue participating in the study during the treatment process, or did not attend the alcohol treatment program. Of these, 637 patients (543 males, 94 females) were qualified to participate in the study [21]. All the patients underwent the standard 10-week alcohol treatment program while in the hospital. While almost all the subjects received the treatment voluntarily, 4 subjects rejected the treatment at admission and were involuntarily hospitalized. After alcohol withdrawal, they accepted and continued the treatment. The program included the treatment of withdrawal symptoms, education regarding the harmful effects of alcohol, cognitive behavioral therapy, occupational therapy, nutrition guidance, and attendance at self-help group meetings. Regarding the prescription of medications for the treatment of alcohol dependence at the time of hospital discharge, two kinds of medications, acamprosate calcium and/or disulfiram, were used. Anti-craving drugs, such as naltrexone and nalmefene, were not available in Japan at the time of study enrollment. The subjects were interviewed regarding their medical and social backgrounds as well as their drinking patterns before admission. Their cognitive functions were assessed using the MMSE during the third week after admission. At around the same time, their psychiatric states were assessed using structured interviews based on the Mini-International Neuropsychiatric Interview (M.I.N.I.) [22]. The assessment screened for and confirmed past and current psychiatric states, such as depressive episodes, panic attacks, psychotic disorder and eating disorder.

After hospital discharge, questionnaires regarding drinking patterns were mailed to the subjects every month until 6 months and then every 2 months until 12 months after hospital discharge. The subjects were asked to complete the questionnaires and to return their responses. If a subject did not return a response, a research assistant phoned the subject and asked him or her to reply to the questionnaire. Subjects who failed to respond three times in a row were regarded as study dropouts. The response rate to the first questionnaire sent one month after discharge was 76.0%. Subjects who were re-hospitalized for the recurrence of alcohol-related problems were interviewed with regard to their drinking between discharge and re-admission, even if they had never returned any of the questionnaires. Therefore, information concerning drinking after discharge was obtained from 80.4% of the subjects.

The primary outcome was the first day of drinking after hospital discharge. Abstinence was defined as continuous non-drinking from the time of hospital discharge. Loss to follow-up was handled as a censored dropout in the survival analyses. The presence or absence of drinking resumption and subsequent drinking patterns were assessed using mailed questionnaires based on the Alcohol Use Disorders Identification Test-Consumption (AUDIT-C), which has been shown to be a reliable and valid self-reported screening instrument [23]. Information on the first day of drinking after discharge was requested in an additional question. To assess the drinking patterns during the follow-up period, the subjects were classified into three groups: “Abstinence” (subjects who remained completely abstinent), “Controlled drinking” (subjects who drank once a month or less and never six or more drinks on one occasion throughout the follow-up period), and “Recurrence” (subjects who drank twice a month or more or who drank six or more drinks on one occasion during the follow-up period).

### Measures

The diagnostic criteria for alcohol dependence in the ICD-10 (International Classification of Diseases, 10th Revision) were checked using a non-standardized psychiatric evaluation performed by specialists skilled in alcohol treatment. The validity of the checked diagnostic criteria was previously confirmed using blood hepatic markers [24].

Current depressive episodes and current or past manic episodes were assessed using the M.I.N.I. Current panic disorder, agoraphobia, social anxiety disorder, obsessive compulsive disorder and posttraumatic stress disorder were also assessed using the M.I.N.I. Subjects who suffered from at least one of these disorders were grouped with those who had any anxiety disorder. Current and past psychiatric disorder and current eating disorder were also assessed using the M.I.N.I. Any psychiatric disorder assessed using the M.I.N.I. was included in the estimate of overlapping psychiatric disorder. Depression and anxiety were also assessed using two self-reported measures: the Zung Self-rating Depression Scale (SDS) [25], and the Beck Anxiety Inventory (BAI) [26].

While the subjects were in the hospital, autistic traits were estimated using the Autism-spectrum Quotient (AQ), which is a self-assessment of one’s personal attitude and communication skills [27]. A score of 33 or greater has been shown to be valid for assessing autistic traits in Japan, although a score of 32 or greater is regarded as indicating a clinically significant level in the United Kingdom [28]. A score of 16 or lower was regarded as the low AQ group in another study conducted in Japan [29]. Subjects who scored 33 or greater were regarded as having ASD traits.

The properties of ADHD were estimated using the Adult ADHD Self Report Scale (ASRS) created by the World Health Organization (WHO) [30]. The reliability and validity of the ASRS have been previously confirmed [31, 32]. The ASRS symptom checklist consists of 18 questions (part A and part B) and measures the frequency of symptoms using a 5-point Likert scale (0 = never, 1 = rarely, 2 = sometimes, 3 = often, 4 = very often). A positive or negative rating for each question is judged according to the 5-point Likert scale. When a subject responds positively to 4 or more of the 6 ASRS questions (part A), the subject is regarded as exhibiting ADHD traits [32]. The total score for the 18 questions (part A and B) using the 5-point Likert scale has also been used to distinguish ADHD subjects from non-ADHD clinical subjects [33, 34]. Since a cut-off of 35 has been shown to be suitable using the ASRS Japanese version [34], subjects with a score of 35 or greater were defined as having ADHD traits in the present study.

ADHD traits were also estimated using interviews based on the Semi-Structured Assessment for the Genetics of Alcoholism-II (SSAGA-II), which is based on the DSM-IV criteria [35]. The reliability and validity of the internalizing symptom sections of the SSAGA have recently been verified [36]. We used the ADHD section of the SSAGA-II in this study. Attention-deficit and hyperactivity/impulsivity were identified when childhood symptoms were present for at least 6 months in at least two settings. If a subject met the criteria for either attention-deficit or hyperactivity/impulsivity as assessed using the SSAGA-II, the subject was regarded as exhibiting ADHD in the present study.

### Statistical analyses

Kaplan-Meier survival analyses were performed to show the time courses of the abstinence rates during the study period. The subjects were classified according to the presence or absence of specific comorbid psychiatric disorders. Differences in the abstinence rates among these groups were then analyzed using both log-rank tests and the generalized Wilcoxon test. To ascertain differences in results arising from the dropout criteria, the sensitivity analyses for the abstinence rates were conducted under the assumption that all the dropouts began drinking at the time of dropout. The survival analyses were processed using JMP 13 Software (SAS Institute).

The average SDS and BAI scores were compared among the three drinking pattern groups. Since the average scores were for continuous variables with normal distributions, *t*-tests were used for the comparisons. Chi-square tests were used to compare the presence or absence of ADHD characteristics among the three drinking pattern groups and to estimate the association with any comorbid psychiatric disorder. These comparisons were processed using an analysis of variance performed with SPSS 19.0 software (IBM).

Multiple Cox proportional hazards analyses using a stepwise method were applied to examine confounding factors. The analyses regarded the duration of abstinence after hospital discharge as the targeted variable. The independent variables included the following factors: gender, age, medications at discharge, education, living status, marriage status, occupation, smoking, current depressive episodes, manic episodes, anxiety disorders, and ASRS score. The hazard ratios of the indicated factors were calculated using SPSS 19.0 software. A value of *p* < 0.05 was regarded as significant throughout the study.

## Results

### Abstinence rates according to mood disorder or anxiety disorder

**Fig. 1:**
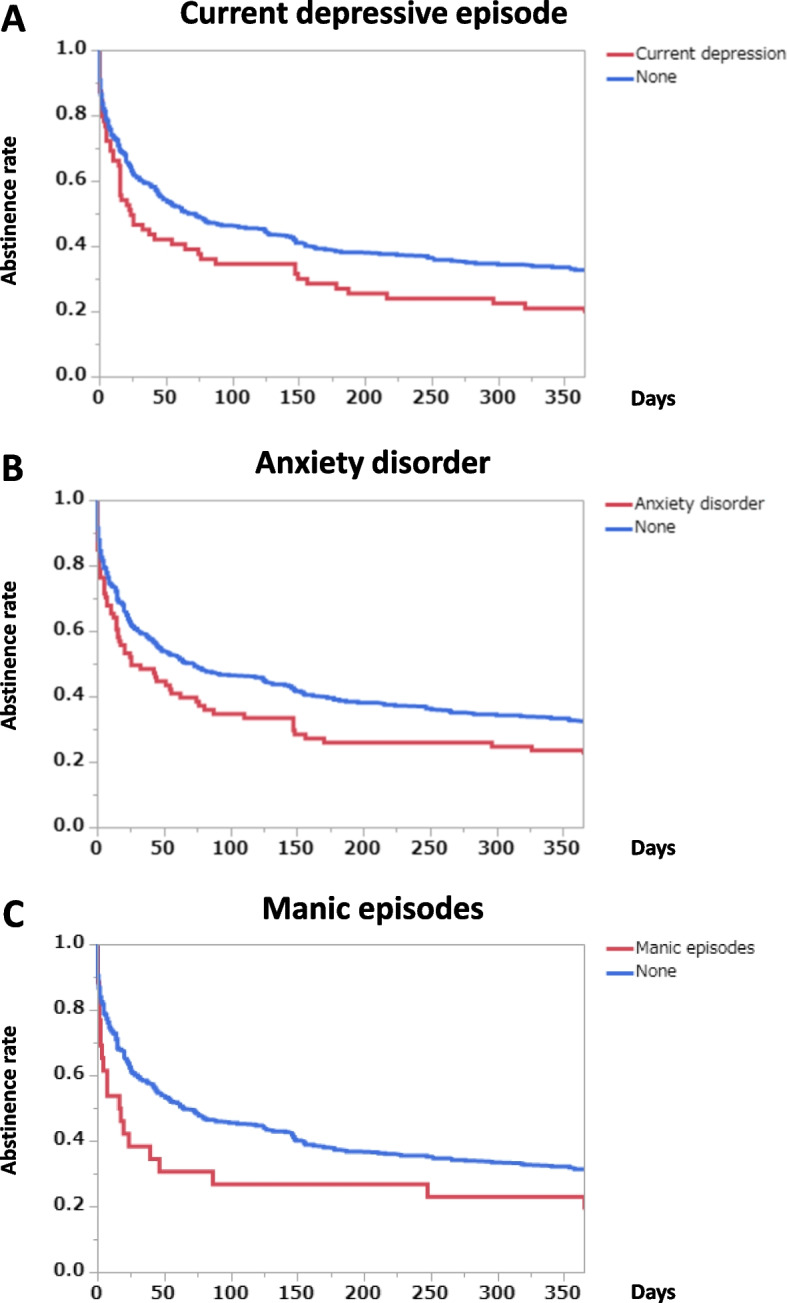
Abstinence rates during the follow-up period according to the presence and absence of psychiatric comorbidities. Graph **A**: Current depressive episode (*p* = 0.0195, log-rank test; *p* = 0.0360, Wilcoxon test). The number of subjects with a current depressive episode was 88, while the number of subjects without a current depressive episode was 454. Graph **B**: Any anxiety disorder (*p* = 0.0214, log-rank test; *p* = 0.0126, Wilcoxon test). The number of subjects with any anxiety disorder was 100, while the number of subjects without any anxiety disorder was 443. Graph **C**: Manic episodes (*p* = 0.0446, log-rank test; *p* = 0.0386, Wilcoxon test). The number of subjects with manic episodes was 35, while the number of subjects without manic episodes was 507

Overall, 16.2% of the subjects had current depressive episodes at the time of admission, while 18.4% had an anxiety disorder at admission. Subjects who reported a current depressive episode at admission had a lower abstinence rate after hospital discharge than those without current depression (Fig. 1A) (*p* = 0.0195, log-rank test). Subjects with any anxiety disorder also had a lower abstinence rate during the follow-up period (Fig. 1B) (*p* = 0.0214, log-rank test).

Overall, 6.5% of the subjects had experienced manic episodes and were suspected of having bipolar I disorder according to the Diagnostic and Statistical Manual of Mental Disorders, Fifth Edition (DSM-5) [37]. Subjects with current or past manic episodes had lower abstinence rates during the 12 months after discharge than the subjects without manic episodes (Fig. 1C) (*p* = 0.0446, log-rank test).

The total SDS and BAI scores were then compared among the three drinking pattern groups after discharge. Although the “Recurrence” group had relatively higher SDS and BAI scores, the subjective assessments of depression and anxiety were not significantly related to the drinking patterns after discharge (SDS, *p* = 0.124; BAI, *p* = 0.680) (Table 2).

**Table 2:** Association of drinking patterns with SDS and BAI scores and ADHD characteristics assessed using the SSAGA-II

		Abstinence	Controlled	Recurrence	Total	r	*p* value
SDS score		44.0 ± 9.6	42.7 ± 7.9	45.8 ± 9.8	–	–	0.124
BAI score		8.0 ± 10.4	7.4 ± 6.9	8.7 ± 8.9	–	–	0.680
ADHD	Presence	27 (25.0%)	6 (5.6%)	75 (69.4%)	108	0.125	0.038
	Absence	105(36.7%)	23 (8.0%)	158(55.2%)	286		
Attention-deficit	Presence	15 (21.1%)	3 (4.2%)	53 (74.6%)	71	0.141	0.013
	Absence	117(36.2%)	26 (8.0%)	180(55.7%)	323		
Hyperactivity	Presence	18 (26.9%)	3 (4.5%)	46 (68.7%)	67	0.078	0.202
	Absence	113(34.8%)	26 (8.0%)	186(57.2%)	325		

### Abstinence rates according to indices of neurodevelopmental disorder

**Fig. 2:**
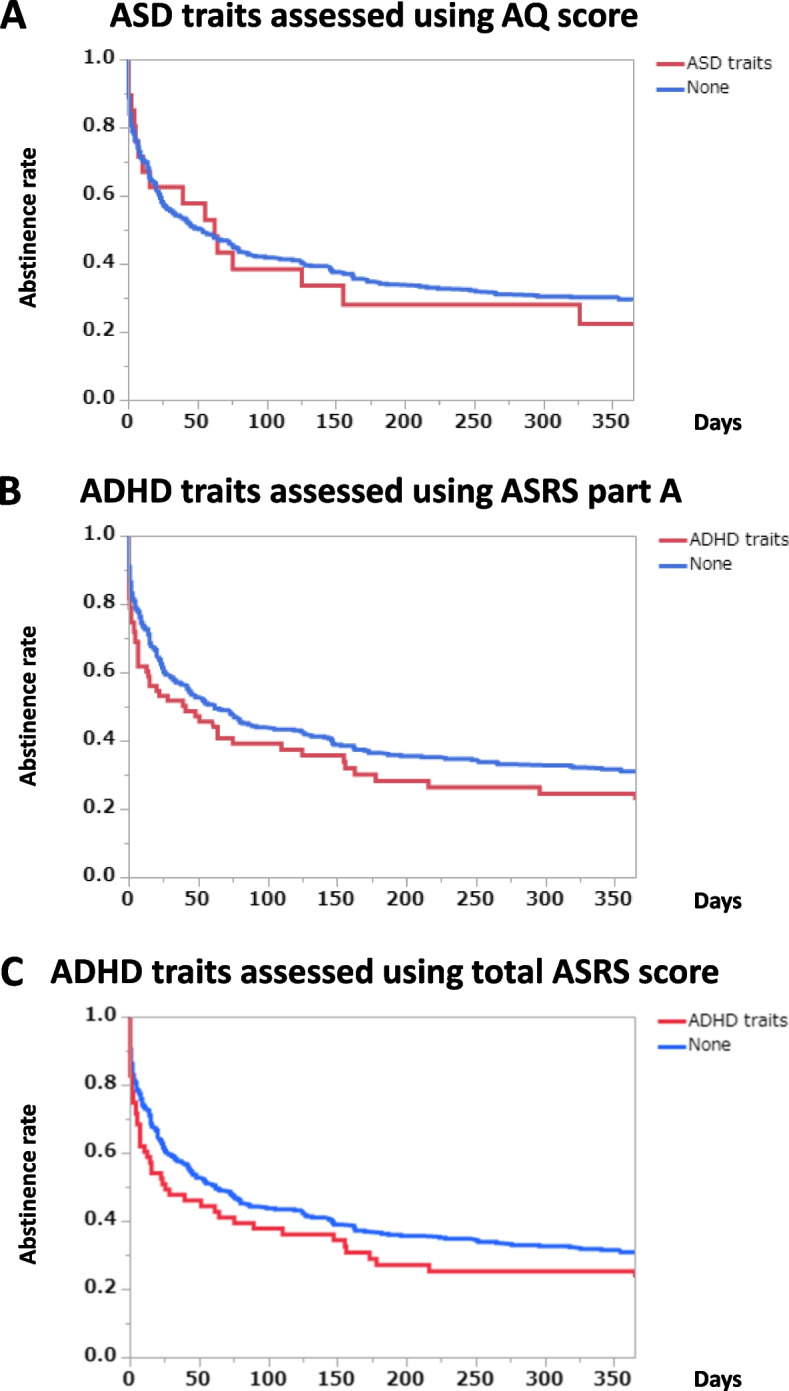
Abstinence rates during the follow-up period according to the presence and absence of ASD or ADHD traits. Graph **A**: ASD traits assessed using the AQ scores (*p* = 0.7537, log-rank test; *p* = 0.9857, Wilcoxon test). The number of subjects with ASD traits was 29, while the number of subjects without ASD traits was 461. Graph **B**: ADHD traits assessed using ASRS part A (*p* = 0.0817, log-rank test; *p* = 0.0296, Wilcoxon test). The number of subjects with ADHD traits was 88, while the number of subjects without ADHD traits was 426. Graph **C**: ADHD traits divided into three classes by using the total endorsed number of ASRS part A and part B (*p* = 0.0995, log-rank test; *p* = 0.0397, Wilcoxon test). The number of subjects with ADHD traits was 76, while the number of subjects without ADHD traits was 438

The presence of autistic traits, as estimated using an AQ threshold score of 33 or greater, did not affect the abstinence rates during the follow-up period (Fig. 2 A) (*p* = 0.7537, log-rank test). Even when the subjects were divided into three groups (low AQ ≤16, moderate AQ, and high AQ ≥33), no differences among the groups were observed (*p* = 0.7204, log-rank test).

ADHD traits were estimated using the ASRS symptom checklist. Subjects with 4 or more positive ratings out of the 6 questions included in part A of the ASRS were regarded as having ADHD traits. Subjects with ADHD traits had lower abstinence rates throughout the follow-up period, compared with those without ADHD traits (Fig. 2B) (*p* = 0.0817, log-rank test; *p* = 0.0296, Wilcoxon test). When the subjects were assessed using the total ASRS score (part A and part B), subjects with a cutoff score of 35 or greater were considered to have ADHD traits. The subjects with ADHD traits had significantly lower abstinence rates after discharge than the subjects without ADHD traits (Fig. 2C) (*p* = 0.0995, log-rank test; *p* = 0.0397, Wilcoxon test).

**Fig. 3:**
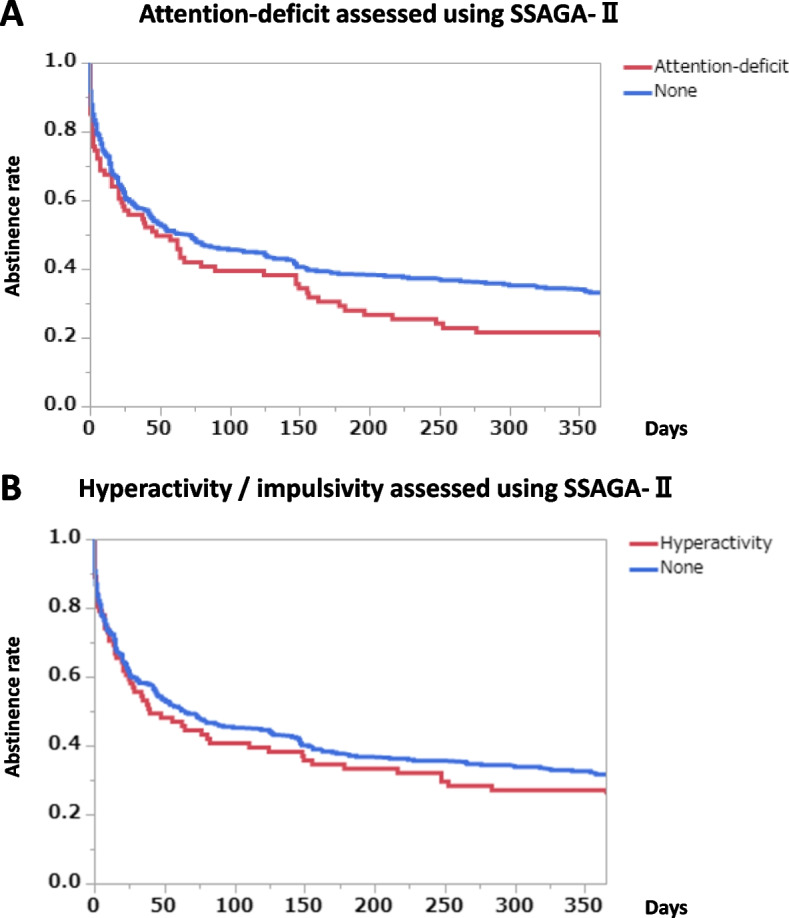
Abstinence rates during the follow-up period according to the presence and the absence of attention-deficit or hyperactivity / impulsivity. Graph **A**: Attention-deficit assessed using the SSAGA-II (*p* = 0.0346, log-rank test; *p* = 0.0451, Wilcoxon test). The number of subjects with attention-deficit was 115, while the number of subjects without attention-deficit was 430. Graph **B**: Hyperactivity / impulsivity assessed using the SSAGA-II (*p* = 0.3314, log-rank test; *p* = 0.3996, Wilcoxon test). The number of subjects with hyperactivity / impulsivity was 102, while the number of subjects without hyperactivity / impulsivity was 441

Using another method, childhood symptoms of ADHD were estimated according to a semi-structured interview based on the SSAGA-II. Subjects who had exhibited attention-deficit during childhood had a significantly lower abstinence rate during the follow-up period, especially during the latter half of the follow-up period (Fig. 3A) (*p* = 0.0346, log-rank test). The subjects who had exhibited hyperactivity and impulsivity during childhood also had lower abstinence rates, but the difference was not significant (Fig. 3B) (*p* = 0.3314, log-rank test). Sensitivity analyses performed under the assumption that all the dropout subjects began drinking at the time of dropout showed unchanged or enhanced results regarding abstinence rates according to the presence or absence of attention-deficit or hyperactivity/impulsivity (Additional file 1: Supplementary Fig. 1 A, 1 B). While the presence of attention-deficit significantly reduced the abstinence rates in the sensitivity analysis (*p* = 0.0019, log-rank test), the presence of hyperactivity/impulsivity hardly affected the abstinence rates during the follow-up period (*p* = 0.5572, log-rank test).

### Correlation between ADHD and drinking patterns

Beyond abstinence rates, the influence of ADHD on drinking patterns after discharge is also a matter of concern. The distributions of subjects with and those without ADHD assessed using the SSAGA-II varied significantly among the three drinking pattern groups (Table 2). Although the effect size was not large, the presence of ADHD significantly influenced the drinking patterns after inpatient treatment (r = 0.125, *p* = 0.038). The analyses were performed separately for the presence or absence of attention-deficit and hyperactivity/impulsivity. The presence of attention-deficit characteristics had a definitive effect on the drinking pattern (r = 0.141, *p* = 0.013), whereas the presence of hyperactivity/impulsivity characteristics did not (Table 2).

### Multivariate cox proportional hazards analyses

Coexisting variables comprised of baseline characteristics, psychiatric comorbidities and neurodevelopmental characteristics were examined using multivariate Cox proportional hazards analyses. The dependent variable targeted the duration of abstinence after hospital discharge. A higher total ASRS score was the sole factor associated with the risk of re-drinking after hospital treatment. Though the hazard ratio of the ASRS score was relatively low (1.023 [95% CI, 1.008–1.039]), the ASRS score was significantly correlated with the re-drinking risk after adjustments for other comorbidities (*p* = 0.003).

### Comorbid psychiatric disorder and ADHD

The effect of any overlapping psychiatric disorder on ADHD should be explored. The correlations between attention-deficit or hyperactivity/impulsivity, as assessed using the SSAGA-II, with the presence of any comorbid psychiatric disorder were both significant (attention-deficit: *p* < 0.001, hyperactivity/impulsivity: *p* = 0.004) (Table 3). The presence of a comorbid psychiatric disorder did not have a significant impact on the abstinence rate of subjects with attention-deficit (Additional file 2: Supplementary Fig. 2A). On the other hand, the presence of a comorbid psychiatric disorder seemed to have an impact on the abstinence rate of subjects with hyperactivity/impulsivity (Additional file 2: Supplementary Fig. 2B). The lowered abstinence rate of subjects with hyperactivity/impulsivity, compared with that for subjects without hyperactivity/impulsivity might be attributable to the presence of the comorbid psychiatric disorder.

**Table 3:** Rates of any comorbid psychiatric disorder in the presence or absence of attention-deficit or hyperactivity/impulsivity characteristics as assessed using the SSAGA-II

		Psychiatric disorder		*p* value
		Presence	Absence	
Attention-deficit	Presence	70 (60.9%)	45 (39.1%)	< 0.001
	Absence	142 (33.4%)	283 (66.6%)	
Hyperactivity/impulsivity	Presence	52 (51.5%)	49 (48.5%)	0.004
	Absence	158 (36.2%)	279 (63.8%)	

## Discussion

This was a prospective study designed to investigate the effects of neurodevelopmental disorders on the course of alcohol use disorder over a 12-month period following inpatient treatment. Subjects with ADHD traits, especially those with attention-deficit characteristics, had significantly lower abstinence rates during the follow-up period. This evidence was confirmed by both subjective self-reports using the ASRS and semi-structured interviews using the SSAGA-II. The presence of ADHD traits influenced not only the abstinence rate, but also the drinking patterns after hospital discharge. Furthermore, the presence of ADHD traits as estimated using the total ASRS score had a considerable influence on the course of alcohol-dependent individuals even after adjustments for confounding factors.

A preliminary study suggested that patients with alcohol dependence and a history of childhood ADHD had a higher rate of recurrence than those without a history of ADHD [38]. However, the study had a small number of subjects, and no significant difference was found. Another study confirmed a diagnosis of ADHD in a sample of patients with alcohol dependence and showed that patients with ADHD relapsed more frequently, even though they were receiving residential treatment [39]. A previous post-hoc analysis targeted subjects with ADHD and SUD and reported that the ADHD subtype did not affect the treatment response, even after controlling for baseline differences [16]. In the present study, however, attention-deficit characteristics, but not hyperactivity and impulsivity, significantly affected the abstinence rates, as shown in the Kaplan-Meier survival analyses (Fig. 3A, B). Sensitivity analyses performed under the assumption that all the dropout subjects began drinking at the time of dropout enhanced these results (Additional file 1: Supplementary Fig. 1A, 1B). The lowered abstinence rates of those with attention-deficit characteristics were not attributable to comorbid psychiatric disorders (Additional file 2: Supplementary Fig. 2A). These results for attention-deficit characteristics probably would have been further amplified if more subjects with attention-deficit had replied to the mailed questionnaires and had been followed after discharge (followed, 19.5% vs. not followed, 28.2%; Table 1). Intense emotions, such as anger, have conventionally been regarded as possible triggers for heavy drinking relapses. While hyperactivity and impulsivity seem to be closely connected with anger cues, only attention-deficit characteristics were significantly associated with drinking after hospital discharge in this study. Hyperactivity and impulsivity are known to decrease, in general, with age. In contrast, attention-deficit can persist late into life [40]. The average ages of the subjects in this study were 55.2 years for men and 46.2 years for women. The persistence of attention-deficit may make it difficult for subjects to sustain a peaceful life and may facilitate re-drinking.

Several studies have reported that individuals with childhood ADHD have a greater likelihood of developing alcohol dependence as they grow older [41, 42]. The prevalence of ADHD among SUD subjects was reportedly 23.1% (95% CI, 19.4–27.2%) in a previous meta-analysis [43]. The prevalence of ADHD among alcohol-dependent patients undergoing long-term treatment was previously reported to be 21.0% when assessed using the Diagnostic Interview for ADHD in Adults (DIVA) [44, 45] and 17.8% when assessed using positive ratings in ASRS part A [44]. In the present study, the prevalence of subjects with ADHD traits was 17.1% when assessed using positive ratings in ASRS part A. The prevalence of subjects with attention-deficit and hyperactivity/impulsivity characteristics were 21.1 and 18.8%, respectively, when assessed using the SSAGA-II.

A high rate of comorbidity is known to exist among ADHD children and adults, and ADHD is commonly accompanied by additional diagnoses [17, 43]. Comorbidity in treatment-seeking SUD patients with and without ADHD was examined in a cross-sectional international study. Seventy-five percent of SUD patients with ADHD had at least one additional comorbid psychiatric disorder, compared with 37% of SUD patients without ADHD [46]. The present study demonstrated similar results. The prevalences of additional comorbid psychiatric disorder among alcohol dependent patients with ADHD and without ADHD were 60.5 and 33.4%, respectively. These results suggest that an interaction between ADHD and alcohol use disorder may lead to additional psychiatric problems.

Reportedly, ASD is not prevalent among SUD subjects [11], partly because of a lack of social skills, reduced access to substance-using peers, and lower novelty-seeking behavior [47]. In contrast, another study reported that subjects with ASD and those with ADHD had almost equal risks for SUD [48]. Because of the differences in the study samples, it is difficult to establish the general prevalence of ASD among SUD individuals [49]. In the present study, 5.9% of the subjects had autistic traits as assessed using an AQ score of 33 or greater, which was previously shown to be a valid threshold in Japan. The AQ score is capable of differentiating between ASD and ADHD even among subjects with comorbid SUD [50]. The present study focused on the course of alcohol-dependent patients after inpatient treatment and found no significant difference between the presence or absence of ASD traits.

Previous studies have shown that alcohol-dependent patients with comorbid mood disorder or anxiety disorder have a poorer course with regard to drinking and abstinence rates [6, 7, 8]. On the other hand, comorbid alcohol-use disorder was significantly associated with persistent depression and/or anxiety disorder [51]. Patients who maintained abstinence or reduced their drinking showed a substantial improvement in their depressive states during the first six weeks [52]. In this study, alcohol-dependent patients with current major depressive episodes at the time of admission had significantly lower abstinence rates during the follow-up period. Similarly, patients with anxiety disorders had significantly lower abstinence rates. Furthermore, patients with manic episodes tended to have lower abstinence rates than those without manic episodes. The result suggests that alcohol-dependent patients with bipolar disorder have likely to have clinically severe conditions and relatively poor courses in terms of drinking, as previously reported [53].

The present study had some limitations. First, the reliability of self-reports regarding drinking using mailed questionnaires is an important factor. The subjects’ answers were promised to be kept confidential and were not to be shared with concerned medical staff members. Although the accuracy of self-reports might be questioned, the reliability of self-reports made by alcohol-dependent individuals has been repeatedly reported [54, 55]. Second, this study targeted treatment-seeking patients, although some patients were forced to attend the hospital by their spouse and family. The subjects were thought to be representative of patients with relatively severe alcohol dependence [56]. Actually, the severities of the subjects were confirmed using the number of ICD-10 criteria that were met [21]. Third, the present study targeted patients who had undergone an intensive 10-week inpatient program. Thus, the results cannot be directly applied to patients treated as outpatients or with brief inpatient treatments. Fourth, the follow-up period after discharge was 12 months. While the follow-up period was not very long, this period was thought to be appropriate for the evaluation of the effectiveness of alcohol treatment. Finally, ADHD traits are more apparent in younger people, and the association between ADHD traits and a poor course in terms of drinking might have been influenced by the ages of the subjects. However, ADHD traits were significantly associated with the re-drinking risk after adjustments for confounding factors, including age.

This study showed an obvious effect of ADHD, especially attention-deficit characteristics, on the drinking patterns of alcohol-dependent patients. Although ADHD is known to be related to the onset of alcohol dependence and frequently overlaps with alcohol dependence [57], this study indicated that ADHD facilitates the relapse of alcohol dependence even after hospital treatment. This facilitation was attributed to attention-deficit characteristics, not hyperactivity or impulsivity. In contrast, ASD traits assessed using AQ scores did not influence the drinking courses after treatment. Since alcohol-dependent patients with ADHD traits can be expected to have a difficult post-treatment course, pharmacological and psychological approaches should be carefully considered. Pharmacological treatment for ADHD might have an impact on the drinking courses of these patients.

## Supplementary Information

**Additional file 1:** **Supplementary Fig. 1.**

**Additional file 2:** **Supplementary Fig. 2.**

## Data Availability

The datasets used and/or analyzed during the current study are not publicly available due to potential invasion of the subjects’ privacy, but are available from the corresponding author on reasonable request.
