# Supplementary material for: Influence of ADHD, especially attention-deficit characteristics, on the course of alcohol-dependent individuals
Source: BMC Psychiatry. 2022 Dec 19;22:803. doi: 10.1186/s12888-022-04455-4 (PMC9762023; doi:10.1186/s12888-022-04455-4)
Supplement: Supplementary file 1 — Additional file 1: Supplementary Fig. 1. [file 12888_2022_4455_MOESM1_ESM.pdf]

A **Attention-deficit assessed using SSAGA-Ⅱ**

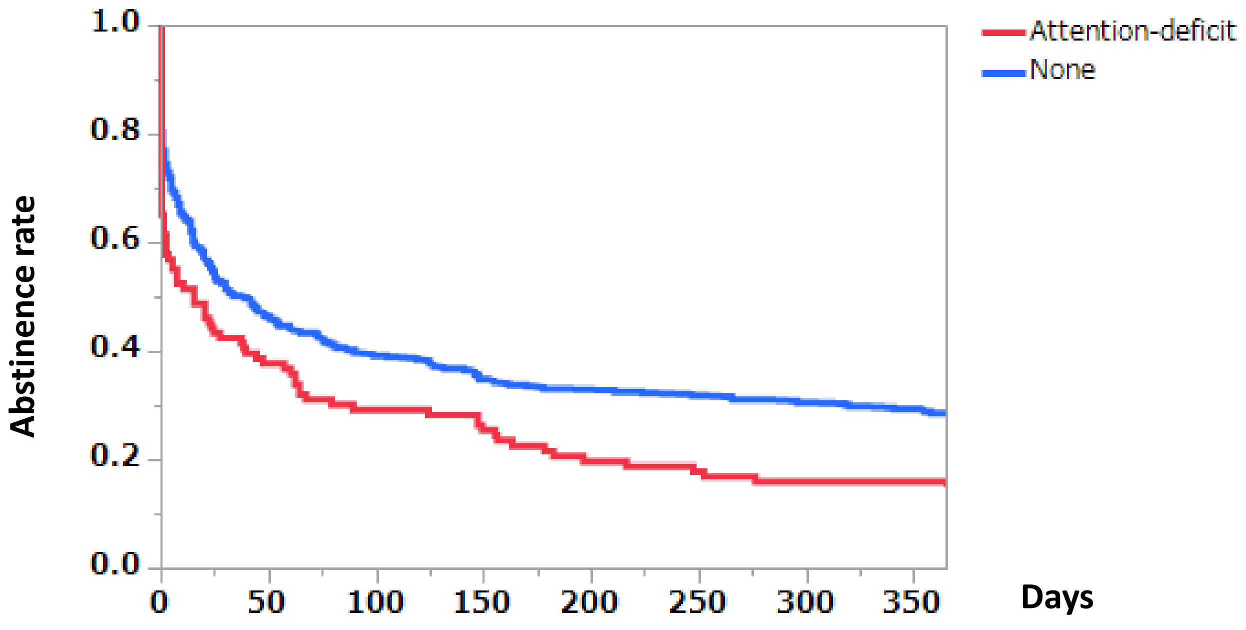

B **Hyperactivity / impulsivity assessed using SSAGA-Ⅱ**

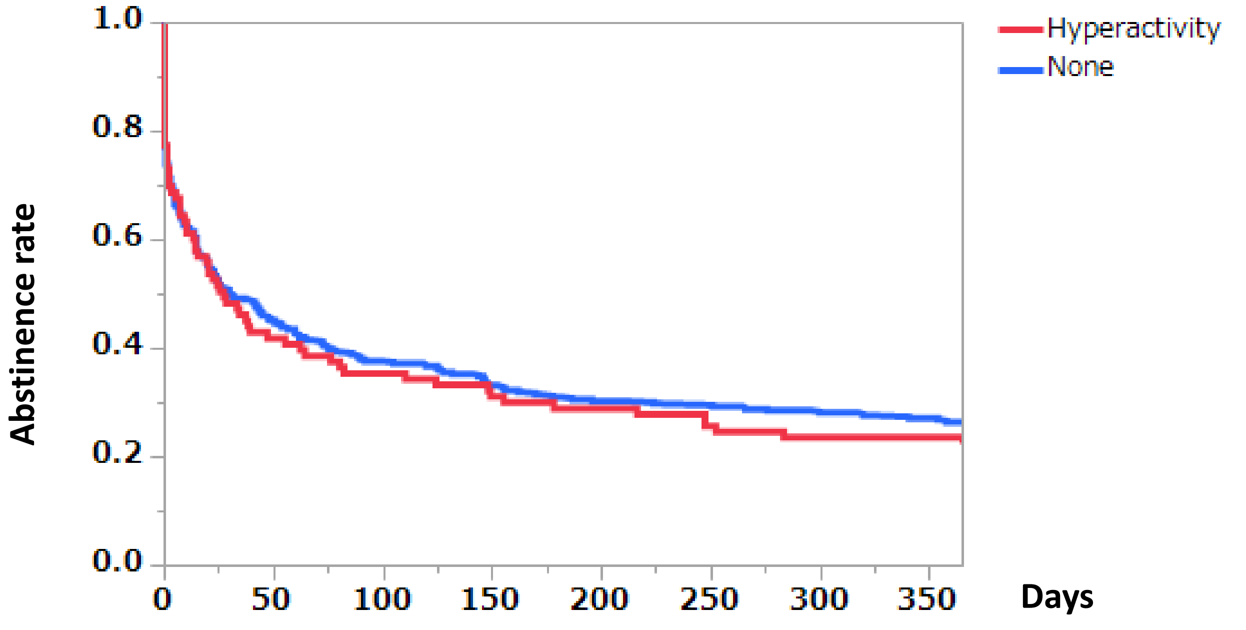

Supplementary Figure 1.
Sensitivity analyses for abstinence rates according to the presence or absence of attention-deficit or hyperactivity/impulsivity
Graph A: Attention-deficit assessed using the SSAGS-II ($p = 0.0019$, log-rank test; $p = 0.0011$, Wilcoxon test). The number of subjects with attention-deficit was 115, while the number of subjects without attention-deficit was 430.
Graph B: Hyperactivity / impulsivity assessed using the SSAGS-II ($p = 0.5572$, log-rank test; $p = 0.7361$, Wilcoxon test). The number of subjects with hyperactivity / impulsivity was 102, while the number of subjects without hyperactivity / impulsivity was 441.
