# Supplementary material for: Influence of ADHD, especially attention-deficit characteristics, on the course of alcohol-dependent individuals
Source: BMC Psychiatry. 2022 Dec 19;22:803. doi: 10.1186/s12888-022-04455-4 (PMC9762023; doi:10.1186/s12888-022-04455-4)
Supplement: Supplementary file 2 — Additional file 2: Supplementary Fig. 2. [file 12888_2022_4455_MOESM2_ESM.pdf]

A **Attention-deficit with or without any psychiatric disorder**

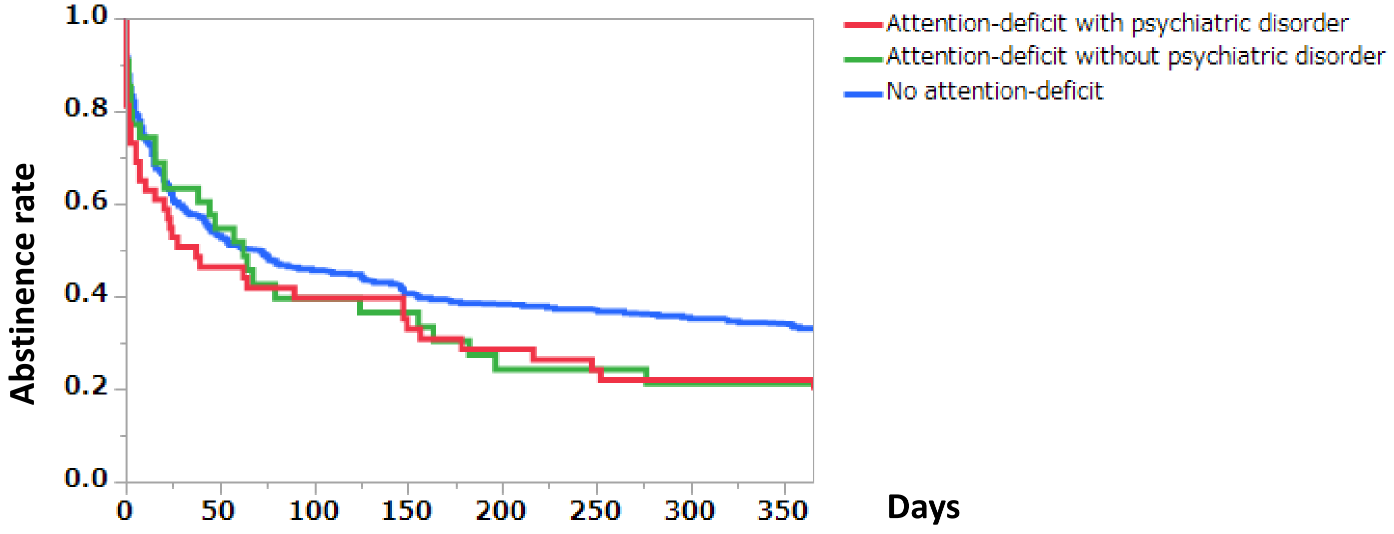

B **Hyperactivity / impulsivity with or without any psychiatric disorder**

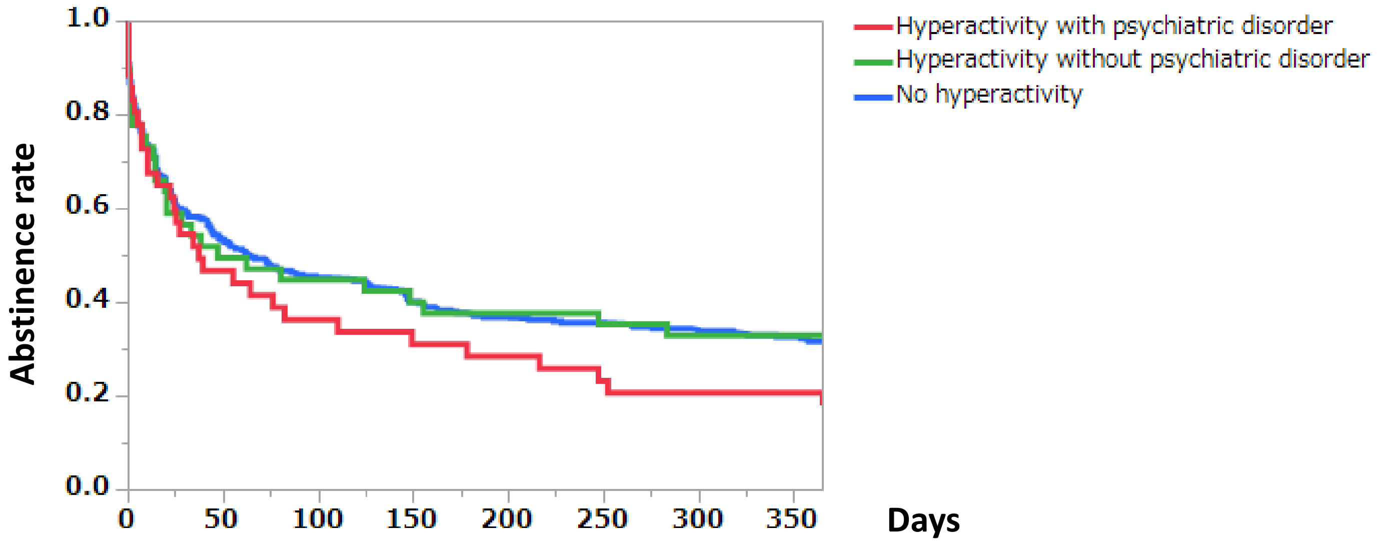

Supplementary Figure 2.
Effects of any comorbid psychiatric disorder on the abstinence rates of the subjects with ADHD characteristics assessed using the SSAGA-II
Graph A: The three groups were compared by the presence or absence of attention-deficit and comorbid psychiatric disorder ($p = 0.0830$, log-rank test; $p = 0.0661$, Wilcoxon test). The numbers of subjects with attention-deficit according to the presence and the absence of comorbid psychiatric disorders were 70 and 45, while the number of subjects without attention-deficit was 430.
Graph B: The three groups were compared by the presence or absence of hyperactivity / impulsivity and comorbid psychiatric disorder ($p = 0.3222$, log-rank test; $p = 0.5659$, Wilcoxon test). The numbers of subjects with hyperactivity / impulsivity according to the presence and the absence of comorbid psychiatric disorders were 52 and 49, while the number of subjects without hyperactivity / impulsivity was 441.
